# Fluorescent Labeling of Helminth Extracellular Vesicles Using an In Vivo Whole Organism Approach

**DOI:** 10.3390/biomedicines8070213

**Published:** 2020-07-14

**Authors:** Anders T. Boysen, Bradley Whitehead, Allan Stensballe, Anna Carnerup, Tommy Nylander, Peter Nejsum

**Affiliations:** 1 Department of Clinical Medicine, Aarhus University, Aarhus 8200, Denmark; anderstb@clin.au.dk (A.T.B.); bradley@clin.au.dk (B.W.); 2 Department of Health Science and Technology, Aalborg University, Aalborg 9100, Denmark; as@hst.aau.dk; 3 Department of Chemistry, Physical Chemistry, Lund University, Lund 210 00, Sweden; anna.carnerup@fkem1.lu.se (A.C.); Tommy.Nylander@fkem1.lu.se (T.N.); 4 Faculty of Veterinary and Agricultural Sciences, The University of Melbourne, Melbourne 3010, Australia

**Keywords:** extracellular vesicles, vesicle labelling, vesicle tracking, helminth, proteomics, Cryo–EM

## Abstract

In the last two decades, extracellular vesicles (EVs) from the three domains of life, Archaea, Bacteria and Eukaryotes, have gained increasing scientific attention. As such, the role of EVs in host-pathogen communication and immune modulation are being intensely investigated. Pivotal to EV research is the determination of how and where EVs are taken up by recipient cells and organs in vivo, which requires suitable tracking strategies including labelling. Labelling of EVs is often performed post-isolation which increases risks of non-specific labelling and the introduction of labelling artefacts. Here we exploited the inability of helminths to de novo synthesise fatty acids to enable labelling of EVs by whole organism uptake of fluorescent lipid analogues and the subsequent incorporation in EVs. We showed uptake of 1,2-dioleoyl-sn-glycero-3-phosphoethanolamine-N-(lissamine rhodamine B sulfonyl) (DOPE-Rho) in *Anisakis* spp. and *Trichuris suis* larvae. EVs isolated from the supernatant of *Anisakis* spp. labelled with DOPE-Rho were characterised to assess the effects of labelling on size, structure and fluorescence of EVs. Fluorescent EVs were successfully taken up by the human macrophage cell line THP-1. This study, therefore, presents a novel staining method that can be utilized by the EV field in parasitology and potentially across multiple species.

## 1. Introduction

Extracellular vesicles, small membranous vesicles that contain a cargo of bioactive molecules are released from organisms spanning all three domains of life [1,2], including parasitic helminths and their hosts [3,4]. The discovery and prediction of miRNAs targeting host genes in EVs released by helminths [5,6] suggest that helminth-derived EVs contribute to host-parasite interactions and may modulate host immune responses, presenting potential translational applications for helminth EVs [3,4,7,8,9]. Central to the study of the biological function of EVs is the accurate determination of cellular uptake or biodistribution. For this, the labelling techniques of EVs are required but this is not facile and post-isolation labelling techniques can introduce artefacts, through the co-isolation of micelles [10,11] or modulation of the EV surface [12] thereby obscuring accurate assessment of the mechanisms, rate of EV uptake in host cells and biodistribution in vivo.

Expression of known EV proteins tagged with fluorescent proteins, such as green fluorescent protein (GFP), provides a specific method of labelling EVs [13]. However, such techniques introducie positive selection of vesicle subtypes, are limited by low fluorescent intensity of EVs, potentially interfere with EV biogenesis via steric hindrance, and are unsuitable for labelling of EVs from primary cells or whole organisms [14].

An alternative method for pre-labelling of EVs was demonstrated by the culture of human bladder cancer cell lines in the presence of the fluorescent lipid analogue, 1,2-dioleoyl-sn-glycero-3-phosphoethanolamine-N-lissamine rhodamine B sulfonyl [15]. Fluorescent rhodamine B-conjugated lipid incorporation within EV membranes during biogenesis allows for direct isolation of fluorescent EVs, in the absence of dye micelles, for subsequent uptake and biodistribution assays. However, to date, the pre-labelling of EVs via fluorescent lipid analogue loading has not been demonstrated for whole organisms.

Parasitic helminths do not synthesise fatty acids and instead acquire lipids and fatty acids from host-tissues, -fluids and/or intestinal content [16]. Indeed, many of the proteins within the excretory/secretory products of helminths include lipid-binding proteins for the appropriation of host-derived lipids [17,18,19]. Importantly, helminths do not metabolise fatty acids for energy production, rather, host-derived lipids are used in the biosynthesis of cell membranes or egg production [20]. Fluorescent lipid analogue uptake has been demonstrated previously for the trematode, *Schistosoma mansoni* [21,22,23]. Therefore, helminths present a highly suitable organism for the assessment of fluorescent lipid analogue labelling strategies of EVs in vivo.

This proof-of-concept study aimed to validate the in vivo uptake of 1,2-dioleoyl-sn-glycero-3-phosphoethanolamine-N-(lissamine rhodamine B sulfonyl) (DOPE-Rho) in nematodes of two different classes; the larval stage of *Trichuris suis* (L1), a whipworm belonging to Enoplea, and *Anisakis* spp. (L3) roundworms belonging to Chromadorea. *T. suis* is a porcine whipworm often employed as a model for *T. trichiura* infection that affects ~290 million people globally [24]. *Anisakis* spp. are zoonotic parasitic nematodes that include *A. simplex* and *A. pegreffii* [25]. *Anisakis* spp. have complex lifecycles including crustaceans and fish as intermediate hosts and marine mammals as the final hosts. They can cause anisakidosis if raw or undercooked fish is consumed, which can cause abdominal pain, nausea, vomiting and potentially anaphylaxis and be fatal in rare cases [26,27]. This study assessed the applicability of this technique for labelling of EVs from *Anisakis* spp. through in vivo uptake of the fluorescent lipid analogue, DOPE-Rho and the functional application of this method in human THP-1 cell uptake studies.

## 2. Experimental Section

### 2.1. T. suis Hatching and Culture

Embryonated *T. suis* eggs were hatched by incubating in 3.3 *v*/*v* % sodium hypochlorite for 2 h at 37 °C with 5% CO_2_ and subsequently exchanged to DMEM (Biowest, Nuaillé, France) with penicillin (100 U/mL) (Sigma Aldrich, St. Louis, MO, USA), streptomycin (100 µg/mL) (Sigma Aldrich, St. Louis, MO, USA) and 1 µg/mL ciprofloxacin (Sigma Aldrich, St. Louis, MO, USA) and incubated for two days at 37 °C with 5% CO_2_.

### 2.2. Anisakis Spp. Harvest and Culture

*Anisakis* spp. were collected from the body cavity of freshly caught herring (*Clupea harengus*) bought from a local vendor. The fish was caught in waters of major fishing areas FAO 27.3.b,c or FAO 27.4.b, which meet the criteria for having both intermediate and definite hosts for *Anisakis* spp. Larvae were washed in PBS (Biowest, Nuaillé, France) (37 °C), followed by incubation in PBS with Anti/Anti solution (Thermo Fischer, Waltham, Massachusetts, USA) containing penicillin (100 U/mL), streptomycin (100 µg/mL) and amphotericin B (250 ng/mL,) at 37 °C for 1 h to prevent microbial contamination.

### 2.3. Larval Uptake of Fluorescent Lipid Analogues in Vitro

Hatched *T. suis* (L1) were divided into three groups, first group designated live uptake, second group designated passive uptake and last group a negative control. The group designated passive uptake were placed on dry ice for 15 min to euthanize the larvae. 4 µM 1,2-dioleoyl-sn-glycero-3-phosphoethanolamine-N-lissamine rhodamine B sulfonyl (DOPE-Rho) (Avanti Polar Lipids, Alabaster, AL, USA) in DMEM were added to the groups except for the negative control to which was just added DMEM. They were then incubated for 2 h. *Anisakis* spp. (L3), were incubated with 0, 1, 4 or 8 µM DOPE-Rho for 5 min or 16 h. Larvae were harvested at the indicated time points, washed three times in PBS prior to fixation in 10 % formalin for *T. suis* and 4% paraformaldehyde (PFA) for *Anisakis* spp. *T. suis* larvae were washed 3 times after fixation and counter-stained with Hoechst-33342 nuclear stain. Larvae were imaged using a Leica DM 2000 LED fluorescent microscope (Leica microsystems, Copenhagen, DK) and images were processed in ImageJ 1.52a (NIH).

### 2.4. Anisakis spp. in Vivo EV Labelling

*Anisakis* spp. L3 larvae were maintained in PBS with Anti/Anti throughout culture at 37 °C and 5% CO_2_. Three *Anisakis* spp. larvae per well were incubated with 0, 1, 4 or 8 µM DOPE-Rho in PBS with Anti/Anti for 16 h. The larvae were then washed with PBS to remove excess dye, given new PBS with Anti/Anti and incubated for 48 h and the supernatant was harvested as conditioned PBS. Dye controls were made in parallel without larvae in the wells and dye solution was directly subjected to EV isolation.

### 2.5. Anisakis spp. Extracellular Vesicle Isolation

EVs from conditioned PBS were isolated by sequential differential centrifugation: 300× *g* for 10 min, 2000× *g* for 15 min, 10,000× *g* for 30 min followed by 110,000× *g* (40.800 RPM TI-50 rotor) (Beckman Coulter, Brea, CA, USA) for 90 min to isolate EVs. EVs were washed once by re-suspending the pellet in PBS and centrifuged once more at 110,000× *g* (40.800 RPM TI-50 rotor) for 90 min. Washed EVs were resuspended in PBS for subsequent characterization and uptake studies. Dye controls were subjugated to the same differential centrifugation. The total protein concentration of intact EVs was assessed using BCA assay as per the manufacturer’s instructions (Thermo Fischer, Waltham, MA, USA). Fluorescence of isolated EVs and dye controls in PBS were assessed using a DS-11 spectrophotometer and fluorometer (DeNovix, Wilmington, DE, USA) with excitation at 525 nm and emission 565–615 nm.

### 2.6. Nanoparticle Tracking Analysis

Extracellular vesicle hydrodynamic radius and concentration were assessed using an NS300 (Malvern Pananalytical, Malvern, UK). Isolated EVs or dye only controls were diluted in filtered (0.2 µm) PBS and analysed immediately hereafter. The following conditions were maintained for all analyses of EVs: particles per frame of 20–100, camera level-15, detection threshold-5 and syringe pump speed of 10 µL/s. Three one-minute videos were captured per sample prior to analysis using Nanosight NTA 3.4.003 software. Hydrodynamic size is given as the mode of 3 measurements in nm (±SEM) and concentration as the mean of 3 measurements in particles/mL (±SEM).

### 2.7. Cryo-Transmission Electron Microscopy

Samples were defrosted and subjugated to short centrifugation to remove aggregates introduced by the freeze-thaw cycle with a Qualitron DW-41. Samples were prepared in an automated plunge freezer system (Leica Microsystems, Wetzlar, Germany). A 4 µL drop of the sample was dispersed on a glow discharged lacey formvar carbon-coated copper grids (Ted Pella Inc, Redding, CA, USA), blotted with a filter paper and then plunged into liquid ethane (approximately −183 °C). The vitrified specimens were thereafter stored in liquid nitrogen (−196 °C) prior to imaging. A Fischione Model 2550 cryo transfer tomography holder was used to transfer the specimen into the electron microscope, JEM 2200FS (JEOL, Tokyo, Japan), equipped with an in-column energy filter (Omega filter), which allows zero-loss imaging. The acceleration voltage was 200kV and energy-filtered images were digitally recorded with a TVIPS F416 camera using SerialEM under low dose conditions with a 25 eV slit in place. Images were processed in ImageJ 1.52a (NIH).

### 2.8. Proteomic Analysis of Extracellular Vesicles

50 µl (>10 µg total protein) from each sample was lysed in 0.1% ProteaseMax in 0.1 M TEAB; sonicated for 2 min in water sonicator and denaturized for 10 min at 60 °C and stored at −80 °C until further processed. Protein concentration was estimated by protein absorbance at 280 nm on a Nanodrop 1000 UV-vis spectrophotometer (Thermo Scientific, Waltham, MA, USA) using the extinction coefficient of bovine serum albumin as a reference (Thermo Scientific Pierce BSA Protein; 2mg/mL). Samples were reduced by incubating with 10 mM tris (2-carboxyethyl) phosphine (Thermo Scientific, Waltham, MA, USA) and 50 mM chloroacetamide (Sigma Aldrich, St. Louis, MO, USA) final concentration at 37 °C for 30 min. The samples were subsequently digested overnight with 1µg of sequencing grade modified trypsin in 0.1 M TEAB (Promega, Madison, WI, USA). Samples were acidified with 0.1% trifluoroacetic acid and reduced by vacuum centrifugation. The reduced samples were dissolved in 30 µL 2 % acetonitrile; 0.1% formic acid; 0.1% trifluoroacetic acid and sonicated for 5 min in a water bath. An aliquot corresponding to 200 ng of tryptic peptides were used for analysis in triplicate (*n* = 3) for quantitative analysis. The samples were separated on a Dionex RSLC UPLC system (Thermo Scientific, Waltham, MA, USA) with uPAC 50 cm analytical column with precolumn (Pharmafluidics, Ghent, Belgium). The samples were loaded at 5 min at 10 uL per min and the mobile phase was ramped over 30 min at a constant flowrate of 700 nL/min from 98% solvent A (0.1% formic acid) and 2% solvent B (0.1% formic acid in acetonitrile) to 45% solvent B in 40 min. Eluted peptides were directly introduced to the coupled ThermoSci QE HF-X mass spectrometer (Thermo scientific, Bremen, Germany) by a picotip emitter for electrospray ionization (New objective, Woburn, MA, USA). The mass spectrometer was operated in positive mode using a data-dependent acquisition method with the following settings: mass range *m*/*z* 375–1200; MS1-scan resolution 120,000; MS2-scan resolution 30,000; isolation window *m*/*z* 1.6 and NCE 28. Peptide hits were searched against *Anisakis* simplex UniProt protein entries (UP000036680; 20,879 entries; released 05/2019) using standard settings in Maxquant v.1.6.12.0 [28]. Proteins of interest were analysed using Blast2Go [29] for BLASTP (NCBI).

### 2.9. Extracellular Vesicle Uptake in Human Macrophage-like THP-1 Cells

THP-1 cells (ATCC, Manassas, VA, USA) were maintained in RPMI-1640 (Biowest, Nuaillé, France) with 10% FBS (Sigma Aldrich, St. Louis, MO, USA) and 0.05 mM β-mercaptoethanol (Sigma Aldrich, St. Louis, MO, USA) at 37 °C and 5% CO_2_. Cells were activated with 100ng/mL phorbol 12-myristate-13-acetate (PMA) (Sigma Aldrich, St. Louis, MO, USA) prior to seeding of 200,000 cells per well in chamber slides for 24 h. Cells were rested in complete media (without PMA) for a further 24 h prior to uptake studies. DOPE-Rho labelled EVs were added at low and high dose, 2 µg and 8 µg total EV protein per well and cells incubated overnight in RPMI with 5% EV-depleted FBS (Thermo Scientific, Waltham, MA, USA). Unlabeled EVs from *Anisakis* spp. were added at a concentration of 12 µg per well as a negative control. Cells were then washed once in PBS, fixed with 10% formalin prior to washing, counterstaining using Hoechst-33342 nuclear stain and mounting in fluoroshield gold (Thermo Scientific, Waltham, MA, USA). Uptake was assessed using a Leica DM 2000 LED fluorescent microscope (Leica microsystems, Copenhagen, DK). Images were processed in ImageJ 1.52a (NIH).

## 3. Results

### 3.1. Fluorescent Lipid Analogue Uptake by T. suis L1 Larvae

**Figure 1:**
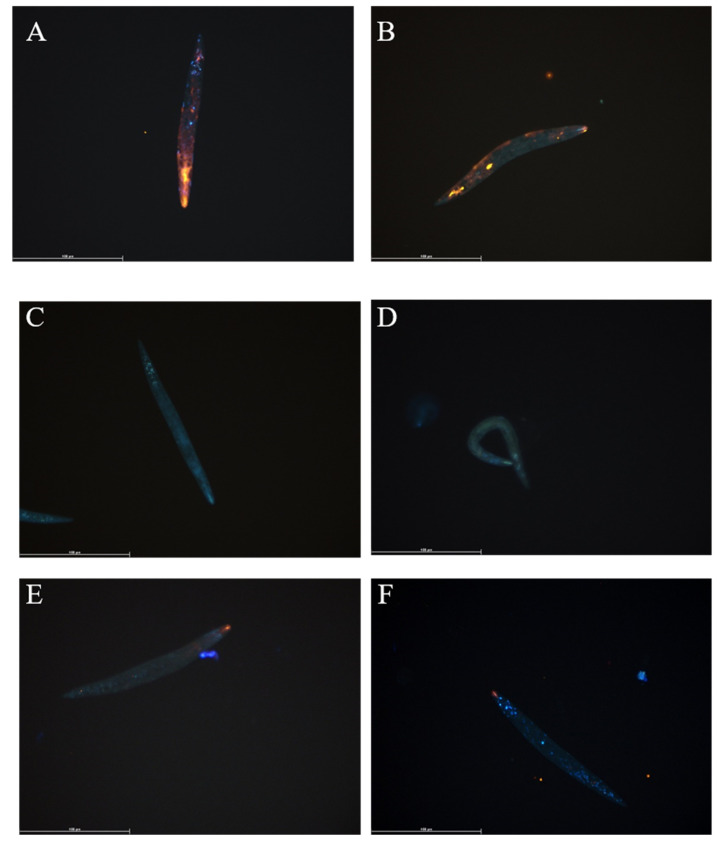
Hatched *T. suis* (L1) cultured for 4 h in the presence of 4 µM DOPE-Rho (**A,B**), control media (**C,D**) and dead *T. suis* (L1) cultured in 4 µM DOPE-Rho (**E,F**) prior to washing, fixation with 4% paraformaldehyde and analysis using fluorescence microscopy. Orange = Rhodamine, Blue = Hoechst nuclear stain, Scale bar: 100 µm.

Hatched *T. suis* L1 larvae were incubated with 4 µM DOPE-Rho in culture media for 2 h prior to washing, fixation, nuclear counterstaining and analysis by fluorescent microscopy. Fluorescent lipid was taken up by live larvae although stain intensity varied amongst larvae, all showed strong fluorescence in the mouth region (Figure 1A,B). No unspecific fluorescence was observed in non-labelled control larvae (Figure 1C,D). To determine if uptake was a passive or active process, larvae were killed by freeze-thaw and incubated for 2 h with 4 µM DOPE-Rho and analysed. The fluorescent signal was detected in the mouth region of dead larvae, albeit at a much lower intensity, but in contrast to live larvae fluorescence was not distributed elsewhere (Figure 1E,F).

### 3.2. Fluorescent Lipid Analogue Uptake in Anisakis spp.

**Figure 2:**
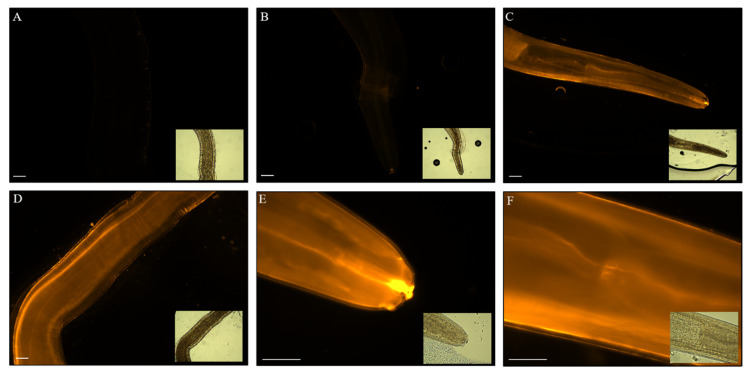
*Anisakis* spp. L3 cultured for 16 h in the presence of 0 µM (**A**), 1 µM (**B**) or 4 µM (**C**–**F**) DOPE-Rho prior to washing, fixation with 4% paraformaldehyde and analysis using fluorescence microscopy. Orange = Rhodamine, Scale bar: 100 µm. Corresponding bright-field images are inset.

DOPE-Rho uptake by *Anisakis* spp., assessed by fluorescent microscopy, was time-dependent with increasing larvae fluorescence at later time-points (Figure S1, Figure 2). Fluorescence of larvae was dependent upon DOPE-Rho concentration with the fluorescent intensity of larvae increasing from 1 to 4 µM DOPE-Rho incubation (Figure 2A–D). However, larvae incubated for 16 h in 8 µM DOPE-Rho showed a reduced fluorescent intensity (Figure S1E,F).

### 3.3. EVs from Anisakis spp. Characterization and Assessment of Labelling

**Figure 3:**
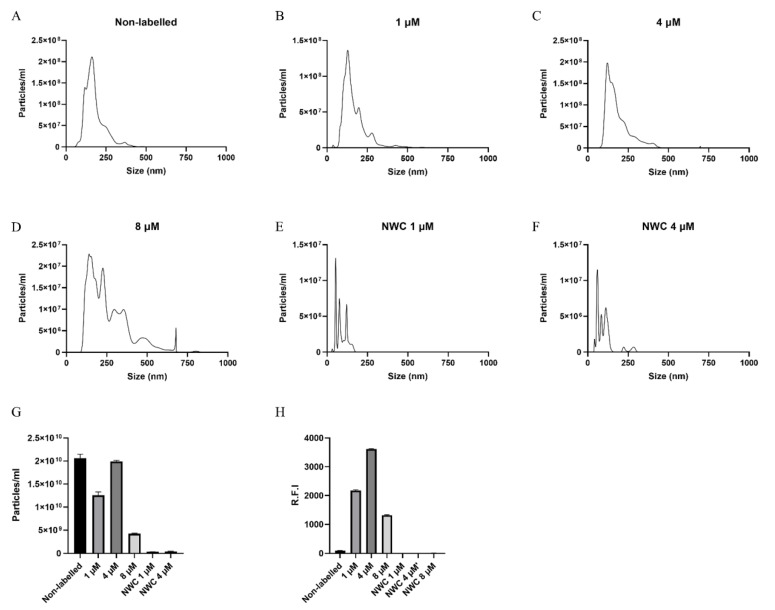
Nanoparticle tracking analysis (NTA) of 100,000× *g* pellets from untreated *Anisakis* spp. (**A**), 1 µM (**B**), 4 µM (**C**) and 8 µM DOPE-Rho incubated *Anisakis* spp. (**D**). NTA analysis of no worm dye control (NWC) 1 µM (**E**) and 4 µM (**F**) 100,000× *g* pellets. Particle number per ml for controls and DOPE-Rho incubated *Anisakis* spp. (**G**). Relative fluorescent intensity (R:F:I) of 100,000× g pellets assessed at excitation 525 nm and emission 565–615 nm (**H**).

Nanoparticle tracking analysis of non-labelled EVs isolated from *Anisakis* spp. culture media showed EVs released had a modal size of 140.5 nm (±7.3 nm) with a mean concentration of 2.06 × 10^10^ (±8.68 × 10^8^) particles/mL (Figure 3A,G). EVs isolated from *Anisakis* spp. incubated with 0, 1, and 4 µM DOPE-Rho had a comparable size distribution, whereas EVs isolated from *Anisakis* spp. Incubated with 8 µM was shifted towards larger particles (Figure 3A–D). Concentration of particles in the EV sample from 4 µM DOPE-Rho incubated larvae was comparable to that of non-labelled with 1.99 × 10^10^ (±2.32 × 10^8^) particles/mL. However, a reduction in particle number was observed for 1 µM and a further reduction for 8 µM DOPE-Rho incubated larvae with 1.26 × 10^10^ (±7.07 × 10^8^) and 4.25 × 10^9^ (±1.14 × 10^8^), respectively (Figure 3G). The dye controls contained particles comparable to what is present in the diluent PBS and were not fluorescent (Figure 3G,H).

### 3.4. Cryo-TEM of Anisakis spp.

**Figure 4:**
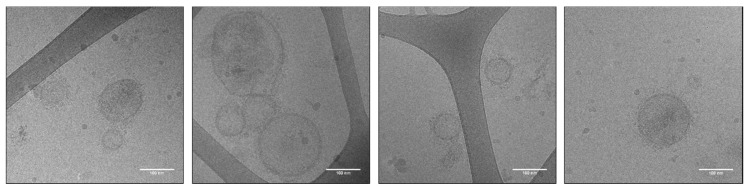
Cryo-TEM images of 1 µM DOPE-Rho labelled *Anisakis* spp. vesicles. Scale bar: 100 nm.

EVs were heterogeneous in size, and most had a corona of surface molecules (Figure 4). The samples were quite dilute with the highest EV number and absence of aggregates, in the labelled samples derived from larvae incubated with 1 µM DOPE-Rho (Figure 4). The EVs derived from larvae incubated with 4 and 8 µM DOPE-Rho contained a high degree of aggregates and few EVs (Figure S2).

### 3.5. EVs from Anisakis spp. Assessed by Quantitative Proteomics to Identify EV Candidate EV Markers

Stringent filtering by inclusion criteria requiring at least two unique peptides + razor in triplicates of both day 3 and day 5 harvested EVs. In the absence of prior knowledge of *Anisakis* spp. EV markers, identified proteins, excluding proteases, were compared to selected helminth EV proteomes in the literature (Table 1). Proteins 14-3-3 and HSP70 that are commonly observed in the proteome of mammalian EVs and also the studies referenced in Table 1 were detected in the EV enriched fraction of *Anisakis* spp. GTP binding proteins such as ras-like-protein 3 and ras-like GTP-binding protein RhoA that are implicated in vesicle biogenesis and transport were also detected in *Anisakis* spp. EV enriched samples. Antioxidant protein superoxide dismutase was also detected in EVs from *Anisakis* spp (Table 1).

### 3.6. Uptake of DOPE-Rho Labelled EVs in THP-1 Cells

**Figure 5:**
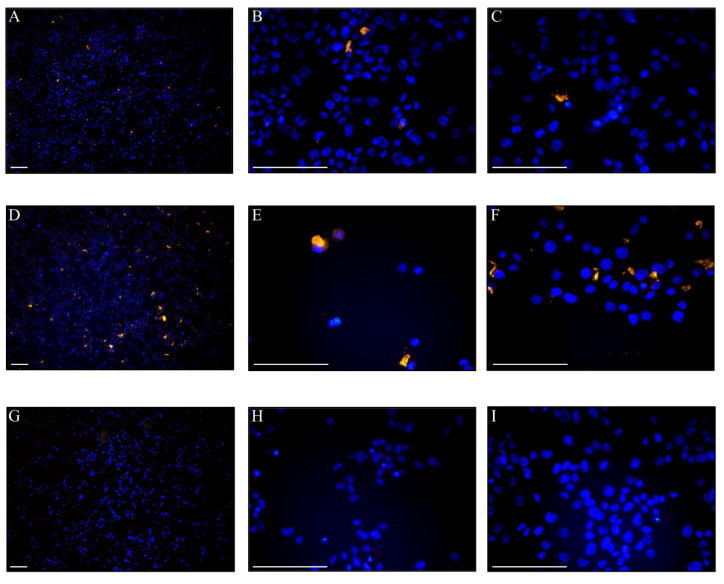
PMA differentiated THP-1 cells incubated overnight with 2 µg (**A**–**C**), or 8µg (**D**,**E** and Figure 1). µM DOPE-Rho labelled EVs. THP-1 cells incubated with 12 µg non-labelled EVs (**G**,**H**) or PBS control (**I**). Orange = Rhodamine labelled EVs, Blue = Hoechst nuclear stain. Scale bar: 100 µm.

To further evaluate this EV labelling method, uptake of labelled *Anisakis* spp. EVs was assessed in PMA activated THP-1 cells. As Cryo-EM determined that the EVs from *Anisakis* spp. labelled with 1 µM DOPE-Rho were the purest, this was used for uptake study. Fluorescent EVs were added at 2 µg or 8 µg (total protein of non-lysed EVs) per well and uptake was assessed using fluorescence microscopy after overnight incubation (Figure 5). Rhodamine fluorescence was detected in a low number of cells incubated with 2 µg DOPE-Rho labelled EVs (Figure 5A–C) with the number of rhodamine positive cells increasing at 8 µg EV treatment (Figure 5D–F). No rhodamine fluorescent signal was observed in cells treated with 12 µg unlabelled EVs (Figure 5G,H) or PBS treated control cells (Figure 5I).

## 4. Discussion

The addition of fluorescent lipid analogue, DOPE-Rho, to culture media was actively taken up in vivo and subsequently incorporated by larvae of nematodes from two differing classes of nematodes. Passive uptake was observed in dead *T. suis* larvae albeit reduced when compared to live larvae. Passive uptake is in line with Furlong et al. (1992) who saw rapid uptake through the outer membrane of *S. mansoni* and accumulation in the oesophageal gland and gut with headgroup labelled phosphoethanolamine [22]. Although the outer surface of nematodes (cuticle) differs from trematodes (tegument) previous studies determined that uptake was mediated via regions of the surface membrane, where lipids can diffuse easily and may explain uptake in dead larvae [35]. Helminths produce and secrete several lipid-binding proteins (LBP) [17,19] that are implicated in the appropriation of host fatty acids and lipids, which could enable lipid-binding to dead larvae. As per the observations of Furlong et al. (1995) in *S. mansoni* cercariae and schistosomula, we also observed accumulation of fluorescent phospholipid at the surface, in the gut [23], and evidence for ingestion of the lipids with staining likely corresponding to the mouth and excretory pore of *Anisakis* spp. While EV subpopulations, such as exosomes and microvesicles, are intensively researched from mammalian sources [1], little is known about the biogenesis and subpopulations of EV derived from parasites. Nevertheless, promiscuous labelling as we have performed would be presumed to label every EV subtype secreted from the parasite.

The modal size of vesicles, 140.5 nm, harvested from *Anisakis* spp. conditioned media is consistent with the size profile of mammalian small EVs [36] and that previously reported for EVs from *A. suum, T. suis, Fasciola hepatica, Brugia malayi* and *Heligmosomoides polygyrus* [6,37,38,39]. Of note, the incubation of *Anisakis* spp. with the fluorescent lipid analogue, DOPE-Rho, did not alter size distribution of EVs from *Anisakis* spp. at 1–4 µM DOPE-Rho concentration. However, the size distribution of vesicles harvested from 8 µM incubated *Anisakis* spp. indicated that the sample was not as pure and suggested altered release or toxicity at this concentration. The concentration of vesicles isolated from non-labelled and 4 µM DPPE-Rho labelled were unchanged, however, a small decrease in vesicles was observed in 1 µM DPPE-Rho EVs. Whilst equal numbers of worms were included per group variations in EV concentration resulting from differences in larvae biomass, and therefore EV production cannot be discounted. However, the greatly reduced number of vesicles in media of 8 µM DOPE-Rho incubated *Anisakis* spp. combined with the reduced lipid uptake in these larvae suggests potential toxicity at this highest concentration. Viability of larvae were only assessed visually, with no reductions in movement observed, but effects on metabolism at this concentration are unknown. Furthermore, the larvae were only kept alive until the harvest of the EVs was performed, prolonging incubation might have revealed further toxicity of the 4 and 8 µMDOPE-Rho concentration.

Using Cryo-TEM we visualized EVs from *Anisakis* spp. with high resolution in their native state with labelling at 1 µM showing the highest purity and EV concentration. Whether the impurities at higher concentrations DOPE-Rho are due to lipid mediating cytotoxicity, and thereby release of dead cells and cuticle, is unknown. The combination of lipid uptake, NTA and cryo-TEM was essential in determining the optimal concentration of DOPE-Rho used in uptake studies and highlights the risks of relying upon single characterisation techniques in EV biology.

To our knowledge, this study presents the first proteomic study of EVs from *Anisakis* spp. and indeed proteins heavily represented in helminth EV proteomes were identified in *Anisakis* spp. EV enriched samples, including 14-3-3 and HSP70 that are almost ubiquitous in proteomic studies of both mammalian [40,41] and helminth EVs [9,42]. Proteases were highly prominent in this analysis, an observation that is consistent with that previously observed for helminth derived EVs [5,6,30,31,32,43]. Given the absence of a priori knowledge of *Anisakis* spp. EV markers and a lack of commercial antibodies targeting previously proposed EV markers of closely related helminths, we propose that the proteomics approach employed and identification of common helminth EV proteins is sufficient to confirm the presence of intact EVs in isolated fractions, when combined with cryo-electron microscopy.

This proof-of-concept study has shown that labelled fluorescent lipid analogues are internalised by nematodes from two classes. Furthermore, using *Anisakis* spp. as a model system, this study has demonstrated that the subsequently released EVs contain fluorescent lipids and their uptake in cells can be assessed by fluorescence microscopy. With the increasing attention on the importance of EVs in host-parasite interaction, this method could not only be used to label all EV subtypes released by nematodes, but may also be applied over a wide range of helminth species. This method could likewise be adapted for the labelling of protozoan EVs, although they both scavenge and synthesize fatty acids de novo, fluorescent fatty acids have been shown to be taken up [44,45,46] and have been employed in the tracking of protozoans intracellularly [46] and trematodes in vivo [21]. The choice of fluorophore-conjugated fatty acid or lipid, uptake duration and concentration should be optimised for each helminth species studied but we propose this may provide a unique method for labelling of EVs in situ for subsequent harvest and use in functional assays that are highly suited to helminth biology.

## Figures and Tables

**Table 1:** Identified *Anisakis* spp. EV proteins cross-referenced to published helminth EV proteomes.

Accession	Protein	Reference ^1^
A0A0M3K8U5	14-3-3	[30,31,32,33]
A0A0M3K9V2	HSP70	[5,6,30,32,33]
A0A0M3IZK3	Tubulin beta	[6,30,31,34]
A0A0M3K9P2	CBN-exc 4	[6,30,31,32] ^2^
A0A0M3KB40; A0A0M3J0M4	Actin	[5,6,33,34]
A0A0M3KFX3	Ras like protein 3	[31]
A0A0M3J8F3; A0A0M3JD57; A0A0M3K4N2; A0A0M3KAB8; A0A0M3KCN6; A0A158PMY7	Maltase glucoamylase	[6]
A0A0M3J727; A0A0M3KA60	Histidine acid phosphatase	[5,6,30,32] ^3^
A0A0M3JAF9	Prostatic acid phosphatase	[5,30,32] ^3^
A0A0M3K4H2	Glutamate dehydrogenase	[6,30,32]
A0A0M3JY91; A0A0M3K219	ATP synthase F1 (alpha + beta subunit)	[6]
A0A0M3JYW4	RAS-like GTP-binding protein RhoA	[6,30]
A0A0M3J718; A0A0M3JZV6	Superoxide dismutase	[32]
A0A0M3JVA0	ADP ribosylation factor 1	[34]
A0A0M3JAH0	Pepsin inhibitor	[32]

**^1^** References for homologous proteins identified in EV proteomes of *H. polygyrus* [5], *F. hepatica* [30,31], *T. muris* [32], *A. suum* [6], *S. mansoni* [33] and *T. circumcincta* [34]. ^2^ Chloride channel exc identified in [31], C-exl 1 identified in [6]. Alternative chloride channels identified e.g CLCN7 [29,30]. ^3^ Identified in references as lysosomal acid phosphatase or acid phosphatase.

## Supplementary Materials

The following are available online at https://www.mdpi.com/2227-9059/8/7/213/s1. Figure S1. Anisakis spp. L3 cultured for 5 min in the presence of 0 µM (A), 1 µM (B), 4 µM (C) or 8 µM (D) 1,2-dioleoyl-sn-glycero-3-phosphoethanolamine-N-(lissamine rhodamine B sulfonyl) prior to washing, fixation with 4% paraformaldehyde and analysis using fluorescence microscopy. Anisakis spp. L3 cultured for 16 h in the presence of 8 µM (E+F)) 1,2-dioleoyl-sn-glycero-3-phosphoethanolamine-N-(lissamine rhodamine B sulfonyl. Orange = Rhodamine, Scale bar = 100 µm. Corresponding bright field images are inset. Figure S2. Cryo-TEM images of 4 µM (A) and 8 µM (B) lipid labelled vesicles. Scale bar 100 nm.
