# Supplementary material for: Fluorescent Labeling of Helminth Extracellular Vesicles Using an In Vivo Whole Organism Approach
Source: Biomedicines. 2020 Jul 14;8(7):213. doi: 10.3390/biomedicines8070213 (PMC7399896; doi:10.3390/biomedicines8070213)
Supplement: Supplementary file 1 [file biomedicines-08-00213-s001.pdf]

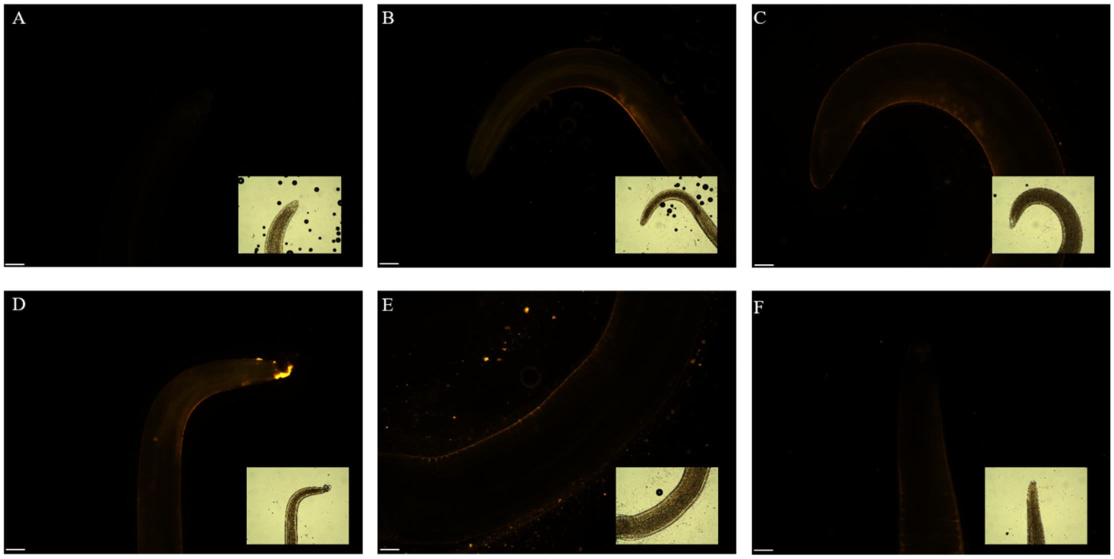

Figure S1. Anisakis spp. L3 cultured for 5 min in the presence of 0 μM (**A**), 1 μM (**B**), 4 μM (**C**) or 8 μM (**D**) 1,2-dioleoyl-sn-glycero-3-phosphoethanolamine-N-(lissamine rhodamine B sulfonyl) prior to washing, fixation with 4% paraformaldehyde and analysis using fluorescence microscopy. Anisakis spp. L3 cultured for 16 h in the presence of 8 μM (**E**+**F**)) 1,2-dioleoyl-sn-glycero-3-phosphoethanolamine-N-(lissamine rhodamine B sulfonyl. Orange=Rhodamine, Scale bar = 100 μm. Corresponding bright field images are inset.

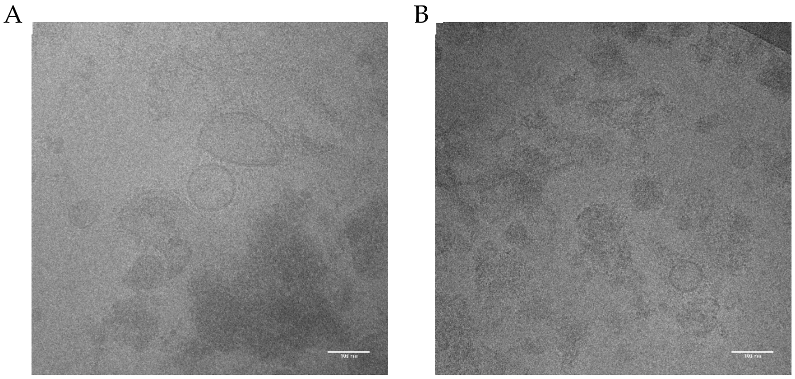

Figure S2. Cryo-TEM images of 4 μM (**A**) and 8 μM (**B**) lipid labelled vesicles. Scale bar 100 nm.
